# Case series of cancer patients who developed cholecystitis related to immune checkpoint inhibitor treatment

**DOI:** 10.1186/s40425-019-0604-2

**Published:** 2019-05-03

**Authors:** Hamzah Abu-Sbeih, Cynthia Nguyen Tran, Phillip S. Ge, Manoop S. Bhutani, Mazen Alasadi, Aung Naing, Amir A. Jazaeri, Yinghong Wang

**Affiliations:** 1 Department of Gastroenterology, Hepatology, and Nutrition, The University of Texas MD Anderson Cancer Center, 1515 Holcombe Blvd., Unit 1466, Houston, TX 77030 USA; 2 Department of Internal Medicine, Baylor College of Medicine, Houston, TX USA; 3 Department of Investigational Cancer Therapeutics, The University of Texas MD Anderson Cancer Center, Houston, TX USA; 4 Department of Gynecologic Oncology and Reproductive Medicine, The University of Texas MD Anderson Cancer Center, Houston, TX USA

**Keywords:** Immune checkpoint inhibitor, Immunotherapy, Adverse event, Gallbladder, Cholecystitis, Immune-mediated cholecystitis

## Abstract

**Background:**

Immune checkpoint inhibitors (ICIs) represent a promising novel class of cancer therapy, but immune-mediated adverse events can complicate ICI treatment. Acute cholecystitis in patients receiving ICI therapy has not been characterized. We aimed to describe the clinical features of patients who developed ICI-related cholecystitis.

**Methods:**

We evaluated a case series of patients at a tertiary cancer center who received ICI therapy and developed cholecystitis, diagnosed by clinical presentation and diagnostic imaging, during 2010–2018. Patients with a history of chronic cholecystitis or other etiologies of acute cholecystitis, such as cholelithiasis, were excluded. A chi-square test was used to compare the frequency of cholecystitis between ICI regimens. Kaplan-Meier and log rank analyses were used to compare survival between subgroups.

**Results:**

Of the 4253 patients who received ICIs in the study period, 25 (0.6%) patients developed suspected ICI-related cholecystitis. Alternatively, of the 31,426 cancer-matched patients who received non-ICI therapy, 72 (0.2%) developed acalculous cholecystitis (*P* < 0.001). Among the 25 included patients, the median time from ICI initiation to cholecystitis was 6 months (range, 0.1–31 months). Fifteen (60%) patients received an inhibitor of programmed death protein 1 (anti–PD-1) or of its ligand (anti–PD-L1) as a single agent, and 10 (40%) patients received an inhibitor of cytotoxic T-lymphocyte associated protein 4 (anti–CTLA-4) therapy alone or combined with anti–PD-1/L1. Anti–CTLA-4 monotherapy was associated with a higher risk of cholecystitis (*P* = 0.006). ICI therapy was discontinued in 20 patients, in three (12%) as a result of acute cholecystitis. Two (8%) patients developed sepsis, and four (16%) had perforation of the gallbladder wall. Five (20%) patients underwent surgical cholecystectomy, and eight (32%) underwent percutaneous drainage. Five (20%) patients were treated with steroids; two of them required surgery. Ten (40%) patients were able to restart ICI therapy. Patients who received a combination of anti–CTLA-4 and anti–PD-1/L1 had more complications of cholecystitis than did patients who received either agent alone (*P* = 0.03).

**Conclusions:**

ICI treatment can result in a clinical condition similar to typical acute cholecystitis in a minority of patients. ICI-related cholecystitis should be managed in a similar fashion to typical cholecystitis. The efficacy of steroids for the treatment of ICI-related cholecystitis is unclear.

**Electronic supplementary material:**

The online version of this article (10.1186/s40425-019-0604-2) contains supplementary material, which is available to authorized users.

## Introduction

Immune checkpoint inhibitors (ICIs) have demonstrated improved survival rates over traditional chemotherapy for a variety of advanced-stage malignancies [1]. Under normal physiologic conditions, the activity of the immune system is regulated by a balance of T-cell activation and tolerance. This balance is mediated by a complex interaction between T-cell receptors and additional signaling molecules. Interactions with cytotoxic T-lymphocyte associated antigen 4 (CTLA-4) receptor or the programmed cell death protein 1 receptor (PD-1) or ligand (PD-L1) negatively regulate T-cell activation and enable tumor evasion from immune detection. Several available monoclonal antibody drugs bind and block these molecules, causing upregulated immune activity and an anti-tumor response [1].

The immune-mediated actions of ICI are not limited to a tumor-related immune response. Increased immune activity can lead to off-target effects in other tissues and organs and produce immune-related adverse events (irAEs) [2]. Gastrointestinal toxic effects are among the most common irAEs, particularly enterocolitis, hepatitis, and pancreatitis [2, 3]. Enterocolitis is the most studied gastrointestinal irAE [4–10]. However, the literature on acute cholecystitis following ICI therapy includes only a few case studies: one due to nivolumab (anti–PD-1) and the other due to avelumab (anti–PD-L1) [11, 12]. Moreover, current treatment guidelines regarding irAEs do not comment on cholecystitis [13–16]. Expanding this knowledge is essential, as the use of immunotherapy is forecasted to increase, and there are several mechanisms that could increase the likelihood of gallbladder inflammation in patients receiving ICI: [1] the presence of liver metastasis with the potential for biliary obstruction, [2] rapid weight loss, which may enhance gallstone formation, [3] altered immunity with potentially increased susceptibility to infections, and [4] risk factors including advanced age, obesity, smoking, and high-fat diet.

The purpose of this study was to facilitate the identification and management of ICI-related cholecystitis, by expanding current knowledge about the clinical features and outcomes, to prevent devastating consequences and sustain ICI therapy.

## Methods

### Study design

We conducted a retrospective case series of patients who received ICI therapy at The University of Texas MD Anderson Cancer Center and developed cholecystitis during January 2010 through June 2018. The study was approved by the Institutional Review Board at MD Anderson. Patients included in the study were at least 18 years old, received ICI therapy under a clinical trial or otherwise, had follow-up abdominal imaging, and presented with cholecystitis. Patients were excluded if they had preexisting chronic cholecystitis or other obvious etiologies of acute cholecystitis, including cholelithiasis or recent endoscopic retrograde cholangiopancreatography. To determine the rate of acalculous cholecystitis in patients who received non-ICI therapy, we used institutional tumor registry database and ICD and SNOMED codes to search for a cancer type-matched cohort who had acalculous cholecystitis.

### Patient characteristics

The medical charts of patients who received ICI therapy and had follow-up abdominal imaging were reviewed to determine ICI regimen. ICIs were categorized as anti–CTLA-4, anti–PD-1/L1, or a combination of both. For included patients, charts were further reviewed for demographic characteristics, prior history of cholecystitis or cholelithiasis, comorbidities, malignancy type and stage, and irAEs. Demographic characteristics included age, sex, race/ethnicity, and body mass index. Comorbidities included smoking, diabetes, hypertension, hyperlipidemia, and ischemic heart disease. Cancers were classified as solid tumors (genitourinary cancer, melanoma, gastrointestinal cancer, or other) or as hematologic malignancies. Solid malignancy staging was assessed in accordance with the American Joint Committee on Cancer’s Cancer Staging Manual, 7th edition [17]. Hematologic staging was not reported owing to its complexity. IrAEs involving organs besides the gallbladder were recorded along with cholecystitis. Use of other chemotherapeutic agents that have been reported to cause cholecystitis was recorded (i.e., azacitidine, idarubicin, or cytarabine).

### Cholecystitis

Clinical data collected pertaining to cholecystitis consisted of presenting symptoms, laboratory results, presence of infection, imaging and histopathologic results, and duration of symptoms. Abdominal pain, nausea, vomiting, diarrhea, and fever were included, representing a spectrum of symptoms of cholecystitis. Typical presentation of acute cholecystitis was defined as right upper quadrant pain with fever, nausea, and/or vomiting. The timing of cholecystitis relative to ICI therapy initiation and the number of ICI infusions before cholecystitis were assessed. The methods of cholecystitis treatment were recorded and consisted of conservative means (intravenous fluids, antibiotics, or steroids) and invasive means (cholecystectomy, percutaneous drainage, or both). Outcomes of cholecystitis, including complications (i.e., sepsis or gallbladder perforation) and need for hospitalization, were determined via review of surgical and consultant notes, microbiology test results, and pathology results. Date of death by any cause or of last follow-up was noted for every patient.

### Statistical analysis

Categorical variables were summarized with frequencies and percentages. Continuous variables were summarized with mean and standard deviation (SD) or median and interquartile range (IQR). Chi-square test was used to compare the frequency of cholecystitis between ICI types and between ICI and non-ICI recipients. Kaplan-Meier curves were used to estimate subgroups’ overall survival, defined as the time from first ICI infusion to death or last follow-up clinical encounter. The log-rank test was used to compare survival rates between subgroups. Analyses were conducted using SPSS Statistics software (version 24.0; IBM, Armonk NY).

## Results

### Patient characteristics

**Fig. 1:**
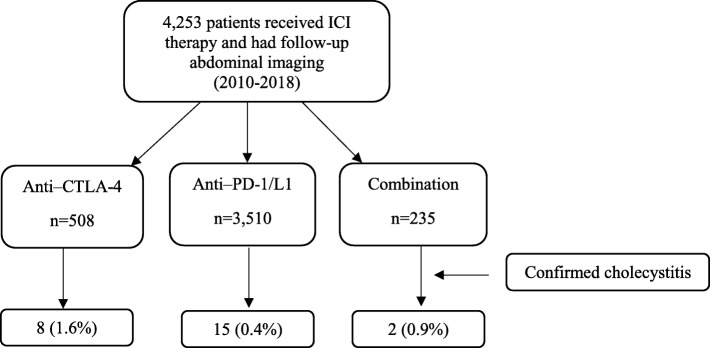
Flowchart of included patients

Of 4253 cancer patients receiving ICI therapy, 25 (0.6%) patients developed cholecystitis and were included in the study group. On the other hand, of the 31,426 cancer-matched patients receiving non-ICI therapy, 72 (0.2%) developed acalculous cholecystitis (*P* < 0.001). Among 4253 patients who received ICI, 44 patients developed cholecystitis due to other reasons (e.g. chronic cholecystitis or acute cholecystitis with cholelithiasis or recent endoscopic retrograde cholangiopancreatography) and were excluded from the study. By ICI type, 508 patients received anti–CTLA-4 monotherapy, 3510 patients received anti–PD-1/L1 monotherapy, and 235 received combination therapy. In the anti–CTLA-4 monotherapy group, cholecystitis developed in eight patients (1.6%), a significantly higher rate than that seen for anti–PD-1/L1 regimens, in which 15 patients (0.4%) in the monotherapy group and two patients (0.9%) in the combination therapy group developed cholecystitis (*P =* 0.006; Fig. 1). Patient characteristics are shown in Table 1. Most patients were white (64%), most were men (60%), and the mean age was 60 years. In our cohort of 25 patients, four patients were given ICI with chemotherapy known to cause gallbladder disease (i.e., azacitidine, idarubicin, or cytarabine). Five patients had liver metastasis at time of ICI initiation that did not progress at time of cholecystitis onset. Other than cholecystitis, skin reactions were the most frequently reported irAE (16%).

**Table 1:** Patient characteristics (n = 25)

Characteristic	Value
Age, years, mean (SD)	60 (11)
Male, n (%)	15 (60)
Race/ethnicity, n (%)	
White	16 (64)
Hispanic	5 (20)
Other	4 (16)
Ever smoker, n (%)	12 (48)
Comorbidities, n (%)	
Diabetes mellitus type 2	6 (24)
Hypertension	18 (72)
Hyperlipidemia	11 (44)
Ischemic heart disease	5 (20)
Body mass index, kg/mm^2^, mean (SD)	28 (5)
Cancer type, n (%)	
Genitourinary cancer	9 (36)
Hematologic cancer	5 (20)
Melanoma	4 (16)
Gastrointestinal cancer	4 (16)
Other	3 (12)
Cancer stage, n (%)^a^	
III	4 (16)
IV	16 (64)
ICI type, n (%)	
Anti–CTLA-4	8 (32)
Anti–PD-1/L1	15 (60)
Combination	2 (8)
ICI given with chemotherapy known to cause gallbladder disease, n (%)	4 (16)
Other irAEs, n (%)	
Skin	4 (16)
Lungs	3 (12)
Endocrine	3 (12)
Colitis	1 (4)
Hepatitis	1 (4)
Other	2 (8)

^a^Available for 20 patients with solid tumors. *SD* standard deviation, *ICI* immune checkpoint inhibitor, *CTLA-4* cytotoxic T-lymphocyte associated antigen 4, *PD-1/L1* programmed cell death protein 1 or its ligand, *irAE* immune-related adverse event

### Clinical characteristics and treatment of cholecystitis

The median time from ICI initiation to onset of cholecystitis was 6 months (IQR, 0.1–31 months), after a median of four ICI infusions (IQR, 1–21 infusions) (Table 2). The presenting symptoms of cholecystitis were abdominal pain in 18 patients (72%), nausea and vomiting in 11 (44%), diarrhea in three (12%), and fever in five (20%). Two patients (8%) had a positive infectious workup at the time of cholecystitis onset, and four patients (16%) received a histopathologic examination of their surgically excised gallbladder showing signs of inflammation. The median duration of symptoms was 5 days (IQR, 3–12 days). Antibiotics were administered to 18 patients (72%), intravenous fluids were administered to 17 (68%), and steroids were administered to five (20%) (Table 3**)**. Fifteen patients (60%) were hospitalized to receive treatment for cholecystitis. Treatment also included percutaneous drainage for eight patients (32%) and surgical cholecystectomy for five (20%); three of them received percutaneous drainage and subsequent cholecystectomy after failure of medical treatments. Histopathologic examination of the gallbladder in these 5 patients who had their gallbladder removed showed unspecific features of active and chronic inflammation, such as erosion and peri-cystic fat necrosis. Ten patients (40%) restarted ICI following the episode of cholecystitis. Cholecystitis symptoms resolved in all patients. No cholecystitis-related deaths were recorded in our cohort.

**Table 2:** Clinical information (n = 25)

Characteristic	Value
Duration of ICI therapy, days, median (IQR)	54 (1–525)
Number of ICI infusions at time of onset, median (IQR)	4 (1–21)
Time from ICI initiation to onset, months, median (IQR)	6 (0.1–31)
Duration of symptoms, days, median (IQR)	5 (3–12)
Peak biochemistry values, median (IQR)	
White blood cell count, cells/L	7.8 (0.7–33.0)
Total bilirubin, mg/dL	1.4 (0.4–36.1)
Direct bilirubin, mg/dL	0.9 (0.2–33.4)
Alkaline phosphatase, IU/L	167 (34–1281)
ALT, IU/L	55 (12–364)
AST, IU/L	47 (16–434)
Cholecystitis clinical presentation, n (%)	
Abdominal pain	18 (72)
Nausea and vomiting	11 (44)
Diarrhea	3 (12)
Fever	5 (20)
Positive infectious workup at time of onset, n (%)	2 (8)
Histopathologic examination performed, n (%)	4 (16)

*ICI* immune checkpoint inhibitor, *IQR* interquartile range, *ALT* alanine aminotransferase, *AST* aspartate aminotransferase

**Table 3:** Treatment and outcomes (n = 25)

Characteristic	Value
Hospitalization, n (%)	15 (60)
Duration of hospitalization, days (IQR)	7 (3–11)
Cholecystitis was the reason to stop ICI treatment, n (%)	3 (12)
Treatment, n (%)	
Intravenous fluid	17 (68)
Antibiotics	18 (72)
Steroids	5 (20)
Surgical cholecystectomy	5 (20)
Percutaneous drainage	8 (32)
Complications, n (%)	
Sepsis	2 (8)
Perforation	4 (16)
Restarted ICI therapy after onset, n (%)	10 (40)
Recurrent cholecystitis symptoms, n (%)	0 (0)
Any-cause death, n (%)^a^	12 (48)

^a^Deaths recorded were unrelated to cholecystitis. *IQR* interquartile range, *ICI* immune checkpoint inhibitor

### Patient characteristics by presence of cholecystitis complications

Cholecystitis-related complications consisted of gallbladder perforation in four (16%) patients and sepsis in two (8%). Both patients who received combination ICI therapy developed cholecystitis complications. A positive infectious workup was found only in patients who had cholecystitis complications. The median duration of symptoms was 9 days in patients who developed complications and 4 days in patients who did not develop complications from cholecystitis (Table 4).

**Table 4:** Characteristics of patients by cholecystitis-related complications

Characteristic	Complications (n = 6)	No complications (n = 19)
ICI type, n (%)		
Anti–CTLA-4	1 (17)	7 (37)
Anti–PD-1/L1	3 (50)	12 (63)
Combination	2 (33)	0 (0)
Months from ICI to onset, median (IQR)	7 (1–10)	5 (0.1–31)
Positive infectious workup, n (%)	2 (33)	0 (0)
Peak biochemistry values, median (IQR)		
White blood cell count, cells/L	11 (5–16)	6 (1–33)
Total bilirubin, mg/dL	1.5 (0.7–3.9)	1.3 (0.4–36.1)
Direct bilirubin, mg/dL	0.9 (0.2–3.5)	1.1 (0.2–33.4)
Alkaline phosphatase, IU/L	169 (34–579)	167 (61–1281)
ALT, IU/L	39 (19–364)	62 (12–296)
Hospitalization, n (%)	5 (83)	10 (53)
Intravenous fluid, n (%)	6 (100)	11 (58)
Antibiotics, n (%)	6 (100)	12 (63)
Surgical treatment, n (%)	5 (83)	6 (32)
Duration of symptoms in days, median (IQR)	9 (5–17)	4 (1–14)
Restarted ICI therapy, n (%)	2 (33)	8 (42)

*ICI* immune checkpoint inhibitor, *IQR* interquartile range, *CTLA-4* cytotoxic T-lymphocyte associated antigen 4, *PD-1/L1* programmed cell death protein 1 or its ligand, *ALT* alanine aminotransferase

### Patient characteristics by presence of typical clinical symptoms

The classically observed cholecystitis symptom of right upper quadrant pain was seen in 18 patients (72%). Patients with typical cholecystitis presentation were more likely to be hospitalized (83% vs. 0%) and receive treatment compared with patients with atypical symptoms (Additional file 1**:** Table S1).

### Patient characteristics and survival by cholecystitis treatment

**Fig. 2:**
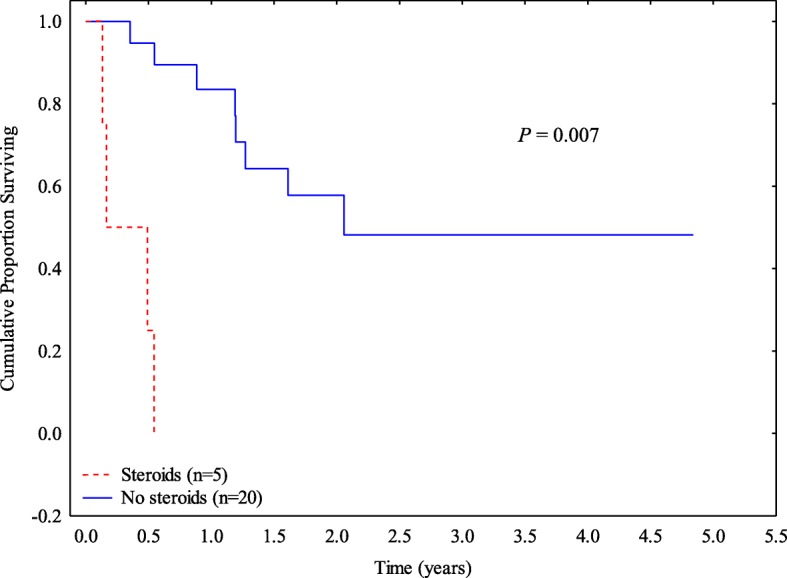
Overall survival by steroid treatment

Treatment with surgery or antibiotics did not accompany any difference in duration of symptoms, duration of hospitalization, resumption of ICI therapy, or death due to any cause (Additional file 1: Table S2). Patients who were treated with steroids had worse survival compared with patients who were not given steroids (*P* = 0.007; Fig. 2). Patients who resumed ICI therapy had longer survival compared with those who did not resume ICI (*P* = 0.016*;* Additional file 1**:** Figure S1). The occurrence of cholecystitis complications did not affect patient survival (Additional file 1**:** Figure S2). Likewise, surgical treatment did not improve survival rates compared with expectant management (Additional file 1: Figure S3).

## Discussion

ICIs are a promising cancer therapy but can lead to irAEs, which can affect any organ, owing to the nonspecific immune upregulation mediated by ICIs. To date, only two cases of ICI-related cholecystitis have been reported [11, 12]. Our case series represents the largest study to date of cancer patients on immunotherapy who developed cholecystitis.

Cholecystitis after ICI therapy is rare, occurring in only 0.6% of patients. This rate was higher than that of patients with corresponding cancer types who received non-ICI therapy (0.2%). We found that cholecystitis occurred significantly more frequently among anti–CTLA-4 recipients than among patients receiving other ICIs (*P* = 0.006), and this trend is similar to that among other irAEs [2]. However, the causality of cholecystitis cannot be attributed to ICI without microscopic confirmation. Hence, future research efforts should focus on establishing the etiology of cholecystitis in relation to ICI therapy. In our cohort, cholecystitis requiring invasive intervention (grade 3 or higher) was seen in 11 patients. In previous reports of ICI-related cholecystitis, only one study has detailed a case of complicated cholecystitis, in which the patient developed sepsis from cholangitis with cholecystitis [11]. In our cohort, six patients had complicated cholecystitis, including sepsis and gallbladder perforation. The incidence of complicated cholecystitis was higher with combination ICI therapy than with ICI monotherapy, which aligns with observations in other studies that increasingly severe irAEs are seen with combination ICI therapy [2].

Our study has many strengths: It is the largest to date of patients with various malignancies who developed cholecystitis after ICI therapy, and it adds to the body of evidence regarding irAEs. It highlights the importance of considering this rare adverse event among differential diagnoses in patients treated with ICIs: resumption of ICI therapy was associated with improved survival in our study. Yet, less than half of our cohort was able to resume ICI therapy following cholecystitis. Thus, the early recognition and appropriate management of ICI-related cholecystitis is vital. The findings of this case series also suggest that traditional management of cholecystitis is appropriate for ICI-related cholecystitis. The role of steroid in the management of ICI-related cholecystitis was unclear in this study.

Patients with malignancy have an increased risk of cholecystitis because they often have multiple risk factors for acute cholecystitis [18, 19]. In our study, many patients had known cholecystitis risk factors, including obesity, smoking, and advanced age. Also, four patients received ICI in combination with a chemotherapy agent known to cause cholecystitis, such as azacitidine. Interestingly, in our cohort, most patients had hematological or genitourinary malignancies despite that majority of patients receiving ICI in the original cohort are melanoma patients. This observation can be explained by the impaired immunity and the use of myelosuppressive therapy in patients with hematological malignancies, where both have been shown to be associated with increased risk of acute cholecystitis [19, 20]. In patients with genitourinary cancers, the frequent metastasis from renal cell carcinoma as well as the use of sorafenib and sunitinib in these patients have been linked with a higher risk of acute cholecystitis [18, 21–23].

The median time to onset of cholecystitis in our study (6 months) was longer than the generally reported timespan of 12 weeks for irAE onset; however, the first onset of irAEs can also occur as long as 1 year after discontinuation of therapy [16]. The clinical presentation of ICI-related cholecystitis was similar to that of traditional acute cholecystitis and included fever, right upper quadrant abdominal pain, nausea, vomiting, diarrhea, leukocytosis, and, for some patients, a positive infectious workup. Some patients had cholecystitis with atypical clinical symptoms but positive imaging findings. Many patients had modest elevations in liver transaminases and had cholestatic laboratory changes, in concordance with case reports [24]. These reports also documented a long latency—4-5 months until cholecystitis onset—which was reflected in our study.

Owing to the rarity of this entity and the current lack of evidence, there is no specific recommendation for management of ICI-related cholecystitis. The general consensus for any irAE recommends supportive care and immunotherapy continuity for mild toxicity and corticosteroids and halting immunotherapy for more severe toxicity. However, corticosteroid therapy is not part of the traditional approach for acute cholecystitis. In our study, patients who received systemic steroids, on the basis of general guidelines for irAEs, had worse overall survival, possibly because of a counter-effect of steroids on ICI therapy. However, we could not delineate the effect of steroid in this study because of the small number of patients receiving steroid and the various possible confounding factors for poor survival rate in these patients, especially being a poor surgical candidate due to impaired functional status and comorbidities. Because the pathogenesis of this form of cholecystitis is presumed to be immune mediated, the potential effect of steroids on survival needs to be clarified in a large-scale study to evaluate whether current treatments for irAEs in general can be appropriate for ICI-related cholecystitis. Future prospective studies validating our findings and investigating the role of steroids in the management of ICI-related cholecystitis are needed.

Despite the strengths of this study, some limitations should be acknowledged. The retrospective design limited the accuracy of the data collection. Second, as MD Anderson is a tertiary care center, some patients were diagnosed and received treatment for cholecystitis at an outside institution. Third, as there are no established guidelines for ICI-related cholecystitis management, many patients who were hospitalized for suspected cholecystitis were treated at the discretion of the treating physician. Fourth, we did not include microscopic examination to delineate the causality of cholecystitis by ICI, and therefore, the association between ICI and cholecystitis still needs to be verified. Last, this study was a case series with a small sample size that did not allow us to perform advanced statistical analysis.

## Conclusion

ICI-related cholecystitis usually presents in a similar fashion to traditional acute cholecystitis and can complicate ICI treatment, leading to its interruption. Our study represents the largest compilation of data regarding this rare entity, which appears to occur more frequently after anti–CTLA-4 therapy compared with other ICI agents. The rate of ICI-related acalculous cholecystitis was higher than that of patients receiving other non-ICI chemotherapy. ICI-related cholecystitis should be managed in a similar fashion to typical cholecystitis. The efficacy of steroids for the treatment of ICI-related cholecystitis is unclear. Clinicians need to be aware of adverse events occurring with ICI use, especially because prompt diagnosis and treatment of these adverse events allow continuation of ICI therapy. Of note, given the lack of response of cholecystitis to steroids, large prospective studies are needed to investigate cholecystitis in relation to ICI therapy and to provide healthcare professionals with evidence-based data for its management.

## Additional file

Additional file 1: **Table S1.** Characteristics of patients by typicality of cholecystitis symptoms. **Table S2.** Characteristics of patients by treatment for cholecystitis. **Figure S1**. Overall survival by the resumption of ICI therapy. **Figure S2.** Overall survival by complications. **Figure S3.** Overall survival by surgical treatment. (DOCX 39 kb)
