# Supplementary material for: Case series of cancer patients who developed cholecystitis related to immune checkpoint inhibitor treatment
Source: J Immunother Cancer. 2019 May 3;7:118. doi: 10.1186/s40425-019-0604-2 (PMC6499962; doi:10.1186/s40425-019-0604-2)
Supplement: Supplementary file 1 — Table S1. Characteristics of patients by typicality of cholecystitis symptoms. Table S2. Characteristics of patients by treatment for cholecystitis. Figure S1. Overall survival by the resumption of ICI therapy. Figure S2. Overall survival by complications. Figure S3. Overall survival by surgical treatment. (DOCX 39 kb) [file 40425_2019_604_MOESM1_ESM.docx]

**Supplemental Table 1.** Characteristics of patients by typicality of cholecystitis symptoms.

| **Characteristic** | **Typical symptoms**  **(n=18)** | **Atypical symptoms**  **(n=7)** |
| --- | --- | --- |
| ICI type, n (%) |  |  |
| Anti–CTLA-4 | 4 (22) | 4 (57) |
| Anti–PD-1/L1 | 12 (67) | 3 (43) |
| Combination | 2 (11) | 0 (0) |
| Number of ICI infusions, median (IQR) | 3 (1-21) | 4 (3-5) |
| Duration of ICI treatment in days, median (IQR) | 36 (1-525) | 61 (1-145) |
| Time from ICI initiation to onset in months, median (IQR) | 6 (0-31) | 6 (2-13) |
| Hospitalization, n (%) | 15 (83) | 0 (0) |
| Intravenous fluid, n (%) | 15 (83) | 2 (29) |
| Antibiotics, n (%) | 16 (89) | 2 (29) |
| Steroids, n (%) | 4 (22) | 1 (14) |
| Surgical treatment, n (%) | 10 (56) | 1 (14) |
| Restarted ICI therapy, n (%) | 7 (39) | 3 (43) |

ICI: immune checkpoint inhibitor; IQR, interquartile range; CTLA-4: cytotoxic T-lymphocyte associated antigen 4; PD-1/L1: programmed cell death protein 1 or its ligand; ALT, alanine aminotransferase

**Supplemental Table 2.** Characteristics of patients by treatment for cholecystitis.

| **Characteristic** | **Antibiotics**  **(n=18)** | **No antibiotics**  **(n=7)** | **Surgical treatment**  **(n=11)** | **No surgical treatment**  **(n=14)** |
| --- | --- | --- | --- | --- |
| Duration of symptoms, days, median (IQR) | 5 (3-13) | 3 (3-3) | 6 (3-17) | 5 (3-6) |
| Duration of hospitalization, days, median (IQR) | 7 (4-13) | 6 (N/A) | 8 (5-12) | 7 (3-32) |
| Restarted ICI therapy, n (%) | 6 (33) | 4 (57) | 3 (27) | 7 (50) |
| Death due to any cause, n (%) | 8 (44) | 4 (57) | 7 (64) | 5 (36) |

ICI: immune checkpoint inhibitor; IQR, interquartile range; N/A, not applicable

**Supplemental Figure 1.** Overall survival by resumption of ICI therapy.

**Supplemental Figure 2.** Overall survival by complications.

**Supplemental Figure 3.** Overall survival by surgical treatment.
